# Growth and reproductive performance by different feed types in fresh water angelfish (*Pterophyllum scalare* Schultze, 1823)

**Published:** 2012

**Authors:** Milad Kasiri, Amin Farahi, Mohammad Sudagar

**Affiliations:** *Department of Fisheries, Faculty of Fisheries and Environmental Sciences, Gorgan University of Agricultural Sciences and Natural Resources, Gorgan, Iran.*

**Keywords:** Growth, Reproductive performance, Angelfish

## Abstract

It is well established that reproduction is sensitive to the state of energy reserves, and that there is a balance between energy homeostasis and fertility. In this view, this study examined the effects of different diets on growth and reproductive performance of fresh water angelfish. Twenty four pairs of angelfish (weighing 3.58 ± 0.24 g) were fed with four types of diets including live earth worm (LEW), dried *Tubifex* (DT), dried *Gammarus* (DG) and prepared granulated feed (PGF), twice a day for 90 days. Reproductive parameters were measured between days 60 and 90. The significant increase in the gonadosomatic index (GSI), fecundity and hatchability brought about by the LEW were demonstrated by the higher number of spawned eggs and hatched larvae. The best growth observed significantly in PGF, and length of larvae was enhanced in this group, consequently. The numbers of dead and deformed fry were lower in the fish fed with PGF and LEW, but there was no significant difference among experimental groups. This study showed that breeders benefit from inclusion of prepared granulated feed and living earth worm during their growth and reproductive stages, and simultaneous using of them for achieving better results is suggested.

## Introduction

Ornamental fish farming is one of the most valuable industries in recent years. Ornamental fish are often referred as living jewels due to their color, shape and behavior. They are peaceful, generally tiny, attractively colored and could be accommodated in confined spaces. The freshwater angelfish (*Pterophyllum scalare*) is one of the South American cichlids that originate from the Guyana and the Orinoco and the Amazon River basins. Angelfish is very popular among aquarists all over the world.^1^

Increasing cost of fish food ingredients (grains, fishmeal, oil cakes, etc.) has made scientists all over the world to look for cheaper and available substitutes. Fishmeal though highly nutritive and palatable is a relatively expensive feed ingredient as compared to other low cost protein rich ingredients such as soybean meal, silk worm pupae, earthworm, etc.^2^ Moreover, in contrast to the culture of edible fish, information on the dietary requirements and feeding ornamental fish is limited.^3^ Nutrition has an important influence on growth and reproductive potential of aquarium fish, and various live feeds have been used for fish rearing. Various live organisms have been used for rearing fish. *Gammarus* is an extremely widespread and abundant crustacean which some of its special characteristics like their food values, high percentage of protein in body mass (more than 40%), vitamins, enzymes, minerals, cartenoid pigments and suitable body size in different life stages make it suitable for feeding fish during different growth stages.^4^ *Tubifex* are excellent food for cultured fish species. The advantages of *Tubifex* in the diet of ornamental fish have been demonstrated. For example, Mandal *et al*.^5^ obtained the best result of growth in fantail guppy (*Poecilia reticulate*) fed with living *Tubifex* than other commercial foods. 

Over the past several years, many people have begun raising earthworms as a source of income or as a means of managing organic waste.^6^ The potential value of earth-worm as a protein source had been established by several authors.^7^^-^^9^ Dynes and Vielma-Rondon *et al*. had shown that not only earthworm could serve as a rich protein source but also as a source of essential amino acids, especially lysine which is limited in many basic foodstuffs and that the amino acid composition of earthworm is very similar to that of fishmeal and potentially superior to that of meat meal.^10^^,^^11^

Artificial diets, which are normally elaborated with dried live organisms processed in different presentations such as flakes, meals or small pellets, are also used. Although it is known that the angelfish accepts artificial diets, lower growth and survival rates of *P. scalare* are commonly obtained when such diets are used as the sole feed.^12^^,^^13^

The latest studies showed that live feeds have various effects on growth and performance of fish.^1^^,^^6^^,^^13^

The purpose of this study was to examine the effect of various diets including living earth worm (LEW), dried *Tubifex* (DT), dried *Gammarus* (DG) and prepared granulated feed (PGF) on the growth and reproductive performance of fresh water angelfish (*Pterophyllum scalare*).

## Materials and Methods

**Experimental fish. **Twenty four pairs of uniform angelfish (weighing of 3.58 ± 0.24 g) aged 11 months were obtained from the Institute of Ornamental Fish Hatchery in Babol (Iran). The feeding trials were conducted on 12 (80×30×40 cm) aquariums. Each aquarium contained one pair of fish. Gentle aeration was provided by air stones. The water temperature was maintained at 28 ± 2 °C during the experiment.

**Experimental diets and feeding regimen** **. **The fish were fed with living earth worm (LEW), dried *Tubifex* (DT), dried *Gammarus* (DG) and prepared granulated feed (PGF) twice a day. Each treatment was performed in triplicate. The experiment was conducted within 90 days. Nutrients composition of trails was showed in Table 1. 

**Table 1:** Nutrient composition of experimental diets (%).

**Diet**	**PGF**	**LEW**	**DT**	**DG**
**Protein**	54.00	62.00	55.00	44.29
**Lipid**	18.00	17.00	14.00	16.90
**Fiber**	1.50	9.00	5.00	-
**Ash**	19.00	5.00	18.00	33.63
**Vitamin**	2.00	-	-	-
**Energy**	4.54	4.65	4.16	3.56

**Measurements and sample analysis** **. **The proximate composition of diets was carried out using the Association of Analytical Chemists Methods. ^14^ Protein was determined by measuring nitrogen (N×6.25) using the Kjeldahl method;^14^ Crude fat was determined using petroleum ether (40–60 Bp) extraction method with Soxhlet apparatus and ash by combustion at 550 °C. Gross energy was determined with an adiabatic bomb calorimeter (PARR 1281, USA). During the experiment, the water quality parameters were monitored. Average value for dissolved oxygen, pH and salinity were 5.70-7.70 mg L^-1^, 6.90-7.80, and 0.10 mg L^-1^, respectively. Dark cycle of 12:12 h was maintained during the feeding trials. Fish from each replicate were measured at the beginning and every 10 days until the end of experiment. Wet weight was determined with an electronic balance (0.01 g precision).

*Weight Gain (WG) = final body weight – initial body weight*

*Specific Growth Rate (SGR) = (* *ln* *W*_t_ *–ln W*_0_ *) ×100 t* ^-1^

Feed Conversion Ratio FCR=Total dry feed consumed (g)Total wet weight gained (g)

Where *ln* is natural logarithm; *W*_t_ and *W*_0_ were final and initial fish weights (g), respectively, and *t* is time (days) between *ln W*_t_ and *ln W*_0_.

**Statistical analysis. **The data obtained from the trial were subjected to one-way analysis of variance (ANOVA) using SPSS version 16.0 (SPSS Inc., Chicago, IL, USA) to test for effects of dietary treatments. When ANOVA identified significant difference among groups, multiple comparison tests among means were performed using Duncan’s new multiple range test. For each comparison, statistically significant differences were determined by setting the aggregate type I error at 5% (*p* < 0.05).

## Results

 The fish were fed by prepared granulated feed (PGF) revealed the best growth; however, there were no significant differences (*p *> 0.05) among the LEW and PGF (Table 2). Accordingly, the best weight gain (3.80 ± 0.43 g) was obtained in this treatment. The proportion of growth in DG was lower than other groups, but there were not any significant differences between DG and DT.

**Table 2:** Nutrient composition of experimental diets (Mean ± SEM).

Parameters	DT	DG	PGF	LEW
Initial weight (g)	3.44 ± 0.24^a^	3.54 ± 0.36^a^	3.75 ± 0.10^a^	3.59 ± 0.22^a^
Final weight (g)	5.93 ± 0.70^bc^	5.59 ± 0.47^c^	7.55 ± 0.39^a^	6.71 ± 0.47^ab^
Weight gain (g)	2.49 ± 0.53^bc^	2.05 ± 0.16^c^	3.80 ± 0.43^a^	3.12 ± 0.25^ab^
SGR	0.99 ± 0.24^bc^	0.79 ± 0.84^c^	1.48 ± 0.13^a^	1.26 ± 0.09^ab^
FCR	2.08 ± 0.22^b^	2.21 ± 0.05^b^	1.54 ± 0.06 a	1.69 ± 0.03^a^

abc Groups with different superscripts differ significantly at *p* <0.05.

**Fig. 1:**
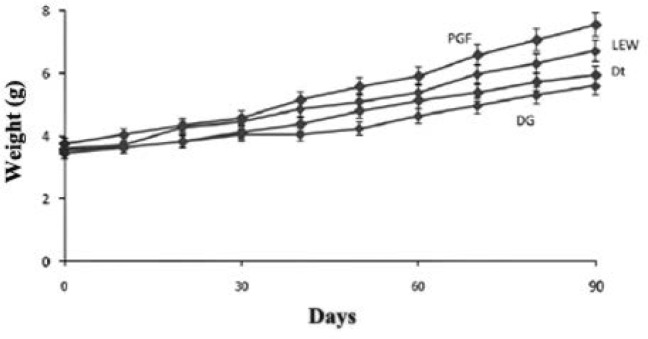
Average weight gain for the four diets evaluated (PGF, LEW, DT and DG).

In the one hand, the greatest SGR was observed in PGF (1.48 ± 0.13). On the other hand, the lowest FCR was obtained in this treatment. Growth trend in PGF and LEW steadily soared (Fig. 1). 

**Fig. 2:**
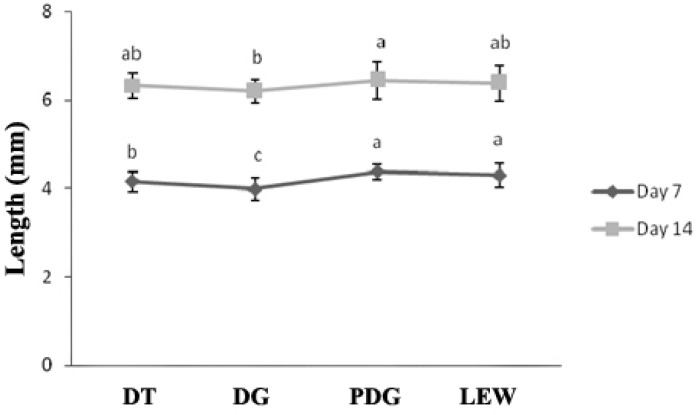
Average fish size for the four diets evaluated

Growth trend in the mentioned groups was better than two others groups. Moreover, DG and DT relatively displayed the same procedure in rearing period. Besides, a significant difference (*p *< 0.05) in the length of larvae on day 7 was observed among treatments (Fig. 2). 

**Table 3:** Reproductive performance of the experimental groups (Mean ± SEM).

**Parameters**	**DT**	**DG**	**PGF**	**LEW**
**Fecundity**	448.33 ± 55.05^b^	361.33 ± 77.10^b^	636.00 ± 127.10^ab^	884.00 ± 114.35^a^
**Fertilization (%) **	77.71 ± 9.32^a^	73.41 ± 10.74^a^	84.62 ± 8.16^a^	90.16 ± 11.73^a^
**Hatching (%) **	60.37 ± 5.60^b^	53.76 ± 4.61^b^	64.67 ± 11.41^b^	76.95 ± 7.39^a^
**Larval survival (%)**	64.56 ± 10.23^a^	62.98 ± 7.99^a^	73.51 ± 9.05^a^	74.10 ± 8.99^a^
**Larval deformity (%)**	2.95±0.45^a^	5.27±5.77^a^	2.61±0.53^a^	2.79±0.26^a^

abc Groups with different superscripts differ significantly at *p* <0.05.

**Fig. 3:**
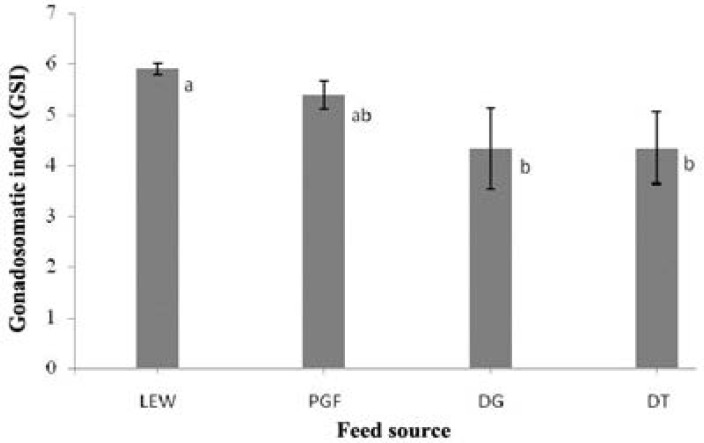
Gonadosomatic index in experimental groups. The typical errors are shown

There was a marked increase for LEW in fecundity, which was significantly different to those of DG and DT (Table 3). There were no significant differences (*p* > 0.05) among the experimental groups in the amount of dead and deformed fry, percentage of fertilization and larval survival. In addition, the percentage of hatching, that was higher in LEW than other experimental groups, illustrated significant promotion in living earth worm treatment. Gonadosomatic index (GSI) was increased drastically in LEW and PGF during the experiment, while this proportion was lower in DG and DT, significantly (Fig. 3).

## Discussion

Several authors have studied the effects of different nutritional diets on the growth, survival and reproductive performance of different fish species.^2^^,^^6^^,^^15^ However, very little information is available on the rearing and early nutritional requirements of ornamental fish. In the present study, four feed sources, namely LEW, DT, DG and PGF were tested to assess the growth, survival and reproductive performance of *Pterophyllum* *scalare.*

It is generally accepted that egg and larval quality are partly controlled by material diet.^6^ Early larval development depends heavily on yolk nutrients delivered through the broodstock diet. Some authors studied the effects of diet on broodstock quality. Limited information is available on the effects of broodstock diet sources on egg and larval quality. The size of larvae was affected by diets (*p* < 0.05), and significant difference was found among treatments in days 7 and 14 (Fig. 2). Equivalently, Devresse *et al.* reported that pronounced improvements in growth was observed when prawn was fed a diet of enriched *Artemia* with medium n-3HUFA.^16^

The highest growth of angelfish was observed in fish fed with PGF and LEW. Prepared granulated feed and live earthworm might have stimulated the feeding behavior of fish and increased the acceptance of feed intake. This could have improved the food intake in two groups. Feed intake of fish depends on size of the prey and predator, quality, density, physical attractiveness and mode of its presentation.^17^ The wriggling movements of large and nutritionally rich prey organisms such as earth worm, *chironomous* larvae and *Culex pipiens* larvae minimize the temporal and energy cost of feeding and maximize growth in *Cyprinus carpio*.^17^ As well as, the best growth was observed in prepared granulated feed group. That could be due to the quality and quantity of protein and other substances essential for growth of angel fish. The fish fed on DG and DT had comparatively lower growth than those fed on PGF and LEW. Reduction in efficiency of diets DG and DT could be related to food quality reduction under drying process.^6^ Longer *et al*. reported that the growth performance of *Macrobrachium dayanum* exposed to different feeds, clearly specified the maximum weight gain and the best FCR and SGR were observed in the prawns exposed to earthworm meal.^2^ Farahi *et al*. recorded significantly better specific growth ratio (SGR) and weight gain for angel fish fed with extruder diet and 50% commercial extruder diet + 50% earthworm.^6^ In this study, the lowest and best FCR value was obtained for the PGF diet, however there was no significant difference between PGF and LEW. Comparably, the best SGR was observed in these groups.

In case of freshwater ornamental fish culture, there is a little information on the nutritional requirements to cover reproductive needs. Morimoto mentioned that for the most of aquatic organisms, the nutritional quality of diets given to broodstock obviously affects biochemical composition of the egg, absolute fecundity and the percentage of hatching.^18^ According to our findings, the best fecundity and hatchability was found in live earth worm diet. It could be linked to the high quality of protein and fatty acid in earthworm. Earthworm lipid has nutritional and medicinal values and it is full of some beneficial substances such as unsaturated fatty acids, ω-3, vit E and cartenoids that can be useful for aquatics health and aquatics foods acceptibility.^19^ Totally in our study, LEW and PGF showed approximately the same positive effects on reproductive performance. This observation was supported by Farahi *et al*. who recorded the same result regarding feeding angel fish by earthworm and extruder diet.^6^ Nevertheless, they asserted that larval survival was greatest in diet which was a combination of earthworm and extruder diet. But our results revealed there was no significant difference among treatments in the survival of larvae.

Gonadosomatic index is of prime importance for understanding gonad development of fish. The gonadosomatic index values (GSI) of angel fish at different feeds were shown in Fig. 3. We observed that the gonadosomatic index were higher in LEW and PGF. Some studies have revealed that high quality of nutrients (especially protein and fatty acids) could improve gonadosomatic index in fish.^20^^-^^22^ We can claim that improvement of gonadosomatic index in LEW and PGF were related to more efficient substances in these diets. 

In conclusion, though other live feeds and diets effectiveness must be studied on angel fish growth and reproductive performance, as far as results of this research are concerned, we strongly believe that application of LEW and PGF can be useful. 

## References

[1] Ortega-Salas AA, Cortes G, Reyes-Bustamante H (2009). Fecundity, growth, and survival of the angelfish Pterophyllum scalare (Perciformes: Cichlidae) under laboratory conditions. Rev Biol Trop.

[2] Langer S, Bakhtiyar Y, Lakhnotra R (2011). Replacement of fishmeal with locally available ingredients in diet composition of Macrobrachium dayanum. Afr J Agr Res.

[3] National Research Council (1993). Nutrient Requirements of Fish.

[4] Mohammadi GH, Khodadadi M, Nasr A, Safikhani H (2010). Fecundity reproductive cycle of a local population of Gammarus Pulex in Sepidan (Fars Province, Iran). Aust J Basic & Appl Sci.

[5] Mandal B, Mukherjee A, Banerjee S (2010). Growth and pigmentation development efficiencies in fantail guppy, Poecilia reticulata fed with commercially available feeds. Agric Biol J N Am.

[6] Farahi A, Kasiri M, Talebi A (2010). Effect of different feed types on growth, spawning, hatching and larval survival in angelfish (Pterophyllum scalare). AACL.

[7] Edwards CA, Niederer A, Edwards CA, Niederer EF ( 1988). The production of earthworm protein.

[8] Orozco MS, Ortega Cerrila ME, Perez-Gil Romo F (1988). Use of earthworm as a protein supplement in diets of rabbits. Arch Latinoam Nutr.

[9] Ortega CMF, Reyes OAL, Mendoza MG (1996). Chemical composition of earthworm (Eisenia fetida and Lumbricus rubellus) silages. Arch Latinoam Nutr.

[10] Dynes RA (2003). Earthworms technology information to enable the development of earthworm production: A report for the Rural Industries Research and Development Corporation Barton, A.C.T.

[11] Vielma-Rondon R, Ovalles-Duran JF, Leon-Leal A (2003). Nutritional value of earthworm flour (Eisenia fetida) as a source of amino acids and its quantitative estimation through reversed phase chromatography (HPLC) and pre-column derivation with o-phthalaldehyde (OPA). Ars Pharm.

[12] Luna-Figueroa J (1999). Influencia de alimento vivo sobre la reproducción y el crecimiento del pez angel Pterophyllum scalare (Perciformes: Cichlidae). Acta Universitaria.

[13] Bahadir Koca S, Diler I, Dulluc A (2009). Effect of different feed types on growth and feed conversation ratio of angelfish (Pterophyllum scalare). J Appl Bio Sci.

[14] (1990). Official methods of analysis of the Association of Official Analytical Chemists.

[15] Hajibeglou AA, Sudagar M (2011). Effect of dietary probiotic level on the reproductive performance of female Platy Xiphophorus maculates. J Anim Vet Adv.

[16] Devresse B, Romdhane MS, Buzzi M (1990). Improved larviculture outputs in the gain freshwater prawn Macrobrachium rosenbergii fed a diet of Artemia enriched with (ω-3) HUFA and phospholipid. World Aquac.

[17] James R, Muthukrishnan J, Sampath K (1993). Effects of food quality on temporal and energetics cost of feeding in Cyprinus carpio (Cyprinidae). J Aquac Trop.

[18] Morimoto H (1994). Effects of maternal nutrition conditions on number, size and lipid content of hydrated eggs in the Japan sardine from Tosa Bay, southwestern Japan.

[19] Hoseini SA, Jalali MA (2010). Use of live food for aquaculture.

[20] Shim KF, Landesman L, Lam TJ (1989). Effect of dietary protein on growth, ovarian development and fecundity in dwarf gourami, Colisa lalia. J Aquac Trop.

[21] Mollah MFA, Sarder MRI, Begum T (2003). Effects of different dietary levels of vitamin E on the breeding performance of Heteroneustes fosslis. Bangladesh J Fish Res.

[22] Begum M, Pal HK, Islam MA (2008). Formulation of quality fish feeds from indigenous raw materials and their effects on growth and maturity of Mystus gulio. J Bangladesh Agril Univ.

